# Decoding tinnitus progression: neurochemical shifts in the anterior cingulate cortex revealed by magnetic resonance spectroscopy

**DOI:** 10.3389/fnins.2025.1551106

**Published:** 2025-02-27

**Authors:** Mengfang Gong, Shuting Han, Yongcong Shen, Yonggang Li, Ji-Sheng Liu, Duo-Duo Tao

**Affiliations:** ^1^Department of Ear, Nose, and Throat, The First Affiliated Hospital of Soochow University, Suzhou, China; ^2^Department of Radiology, The First Affiliated Hospital of Soochow University, Suzhou, China

**Keywords:** tinnitus, GABA, anterior cingulate cortex, recent onset, chronic

## Abstract

**Background:**

Tinnitus persists as a significant public health challenge with elusive neurochemical underpinnings. Emerging evidence implicates dysregulated excitatory-inhibitory neurotransmission in the anterior cingulate cortex (ACC), a limbic-auditory hub governing tinnitus salience. This study investigates dynamic ACC neurochemical changes during tinnitus progression.

**Methods:**

Using single-voxel magnetic resonance spectroscopy (MRS), GABA+/creatine (Cr) and Glx (glutamate+glutamine)/Cr ratios were measured in the ACC of 16 recent-onset (RO; <6 months), 22 chronic (CH; ≥6 months) tinnitus patients, and 26 healthy controls (HC). Tinnitus severity was assessed via tinnitometry and Tinnitus Functional Index (TFI).

**Results:**

RO patients exhibited significantly reduced ACC GABA+/Cr compared to CH and HC groups (*p* < 0.05), while CH and HC showed no differences. GABA+/Cr positively correlated with tinnitus duration across patients (*r* = 0.364, *p* = 0.025). Although Glx/Cr did not differ between groups, elevated Glx/Cr associated with higher tinnitus pitch-matching frequencies (r = 0.421, *p* = 0.008) and emotional distress (TFI-E; *r* = 0.370, *p* = 0.022), though these findings did not survive multiple comparison correction.

**Conclusion:**

Early tinnitus is characterized by ACC GABAergic deficits, while chronicity features normalized GABA+/Cr levels—suggesting compensatory neuroplastic restoration of inhibition over time. Glutamatergic activity may modulate perceptual and emotional dimensions of tinnitus. These phase-specific ACC neurochemical shifts highlight potential therapeutic targets for arresting tinnitus progression. Longitudinal studies are warranted to validate temporal dynamics.

## 1 Introduction

Subjective tinnitus (hereafter referred to as tinnitus), the perception of sound without an external source, is a significant public health issue. Affecting approximately 10–15% of the global population (Mccormack et al., 2016), it results in severe distress for 1–2% of individuals, severely impairing quality of life by disrupting sleep, inducing anxiety, and reducing productivity (Messina et al., 2022; Pattyn et al., 2016). Despite its widespread prevalence, the underlying mechanisms remain poorly understood (Eggermont and Roberts, 2015; Langguth et al., 2019). While peripheral auditory dysfunction often initiates tinnitus, its persistence and emotional salience are increasingly attributed to maladaptive central plasticity (Rauschecker et al., 2010).

Recent advances highlight the anterior cingulate cortex (ACC) as a critical hub in tinnitus pathophysiology. Unlike auditory regions that primarily encode phantom sound characteristics (e.g., pitch/loudness), the ACC’s unique position as a limbic-auditory interface enables it to gate tinnitus perception through three mechanistically distinct roles: (1) Emotional Salience Encoding. ACC’s connectivity with the auditory cortex influences the salience of auditory stimuli (Fan et al., 2024). Altered functional connectivity between the ACC and auditory cortex has been observed in tinnitus patients, and these changes correlate with increased distress and prolonged symptom duration (Chen et al., 2017; Chen et al., 2018; Fan et al., 2024). Moreover, experimental models suggest that tinnitus-related hyperactivity in the ACC may involve increased extracellular glutamate levels and spontaneous firing rates, further supporting the ACC’s involvement in tinnitus perception and distress (Fan et al., 2021). (2) Noise Cancellation. Dysfunctional ACC-mediated filtering of irrelevant auditory signals exacerbates tinnitus awareness (Song et al., 2015). (3) Adaptive Plasticity. ACC hyperactivity in recent-onset tinnitus precedes downstream network reorganization (Vanneste et al., 2011).

Evidence also suggests that the ACC’s role in tinnitus varies across stages of the condition. In RO tinnitus, increased activity in the dorsal ACC correlates with decreased functional connectivity between auditory and non-auditory regions, indicating neural reorganization during the early phases (Vanneste et al., 2011; Vanneste et al., 2024). In contrast, chronic (CH) tinnitus is associated with disrupted connectivity patterns across broader networks, including the prefrontal cortex, auditory cortex, and the default mode network, often linked to tinnitus duration and emotional distress (Chen et al., 2018; Vanneste and De Ridder, 2013).

These stage-specific ACC dynamics suggest its neurotransmitter milieu may orchestrate tinnitus chronification. Previous studies found that neurotransmitter imbalances, particularly those involving gamma-aminobutyric acid (GABA) and glutamate, have been increasingly identified as crucial factors in the development and persistence of tinnitus (Middleton et al., 2011; Sedley et al., 2015; Brozoski et al., 2012; Zhang et al., 2021; Isler et al., 2022). For instance, reduced GABAergic inhibition in the dorsal cochlear nucleus is associated with hyperactivity, which is a hallmark of tinnitus-related neural dysfunction (Middleton et al., 2011). Additionally, glutamate, the predominant excitatory neurotransmitter, is involved in excitotoxicity and synaptic plasticity, both of which can lead to abnormal auditory signaling when disrupted (Schaette and Kempter, 2012; Saidia et al., 2024; Wenthold et al., 1997). The balance between these neurotransmitter systems, especially their excitatory–inhibitory equilibrium, is critical for maintaining auditory homeostasis, and its disruption may contribute to tinnitus chronification (Brozoski et al., 2012; Richardson et al., 2012; Eggermont, 2005). However, prior studies focused narrowly on auditory pathway neurotransmitter imbalances or global GABA/glutamate measures, leaving the ACC’s neurochemical trajectory for tinnitus, especially the neurotransmission evolution from RO to CH tinnitus, unexplored. Investigating these changes may offer insights into the dynamic nature of tinnitus pathophysiology, help identify biomarkers for its progression, and guide the development of targeted therapeutic interventions.

Investigating ACC neurochemistry requires a method capable of non-invasive measurement of GABA and glutamate *in vivo*. Magnetic resonance spectroscopy (MRS) was selected as the primary method. Unlike other techniques (e.g., PET or invasive microdialysis), MRS provides a direct, region-specific assessment of GABA. It has been widely validated in GABA/Cr levels measurement in ACC (Chen et al., 2023; Peek et al., 2021). This study seeks to address these gaps in knowledge by using MRS to measure GABA and glutamate levels in the ACC of individuals with RO and CH tinnitus. So far as we know, this study is the first one to explore ACC neurochemistry in tinnitus patients, which aims to clarify the neurobiological mechanisms underlying tinnitus perception and its chronification by correlating neurochemical findings with psychoacoustic and self-reported severity measures.

## 2 Materials and methods

### 2.1 Participants

Participants were recruited according to the following criteria:

Inclusion criteria (tinnitus group): (1) Adults (18–65 years) with subjective unilateral or bilateral tinnitus. (2) Clinically normal hearing (pure-tone average [PTA] ≤25 dB HL at 0.5–4 kHz). (3) Tinnitus classified as either: Recent-onset (RO): Duration <6 months; Chronic (CH): Duration ≥6 months.

Exclusion criteria (all participants): (1) Objective tinnitus, Meniere’s disease, sudden sensorineural hearing loss, or structural abnormalities (e.g., acoustic neuroma, middle/inner ear pathologies). (2) History of major neurological/psychiatric disorders (e.g., stroke, dementia, schizophrenia) or systemic conditions affecting CNS function (e.g., renal failure, untreated hypertension). (3) Current use of medications influencing GABA/glutamate neurotransmission (e.g., benzodiazepines, anticonvulsants, ototoxic drugs) within 30 days prior to enrollment. (4) Active psychiatric comorbidities (e.g., major depressive disorder, generalized anxiety disorder) diagnosed via clinical interview. (4) Inability to comply with protocol requirements (e.g., severe claustrophobia, non-removable metal implants, language barriers).

Healthy controls (NC) met equivalent audiometric and medical criteria but reported no history of tinnitus. Sixteen participants with RO tinnitus (6 females, 10 males), 22 participants with CH tinnitus (5 females, 17 males), and 26 healthy controls (normal control, NC) (10 females, 16 males) were recruited for the study. The mean age was 36.3 ± 9.4 years for the RO tinnitus group, 37.2 ± 11.2 years for the CH tinnitus group, and 39.2 ± 11.9 years for the non-tinnitus group. The pure-tone average (PTA) threshold across 500, 1,000, 2000, and 4,000 Hz was 15.9 ± 4.8 dB HL for the RO tinnitus group, 13.5 ± 5.8 dB HL for the CH tinnitus group, and 16.2 ± 5.2 dB HL for the non-tinnitus group. All participants were recruited from the First Affiliated Hospital of Soochow University, Suzhou, China. All procedures followed the review and approval of the Ethics Committee of Soochow University, Suzhou, China (approval number: 2021-111, June 7, 2021). All participants provided verbal and written consent before participating in the study, and all privacy rights were respected.

### 2.2 Tinnitus severity measures

Tinnitus severity was assessed in the tinnitus group using a visual analog scale (VAS; Shin et al., 2022) and the Tinnitus Functional Index (TFI; Meikle et al., 2012). For VAS, participants were asked to mark their tinnitus severity on a 10-cm line, with 0 representing no tinnitus and 10 representing the worst tinnitus imaginable. Cartoon expressions (e.g., smile, neutral, pain, and extreme pain) were provided above the line to illustrate the range of tinnitus severity. The TFI consists of eight subscales: intrusive, sense of control, cognitive, sleep, auditory, relaxation, quality of life, and emotional (E). The TFI includes 25 questions, each with a response scale of 0–10, except for two items that use a scale of 0–100% with a 10% increment, with higher scores indicating more severe tinnitus issues. The total score is calculated by summing all responses, with a maximum score of 10 for each question, resulting in a possible total score of 250. The total score was then divided by the number of valid answers, multiplied by 10, to obtain a score out of 100. The raw VAS and TFI data are available in the Supplementary Data Sheet 1.

### 2.3 Tinnitometry

Pitch matching (PM) was performed using pure-tone stimuli delivered through headphones, with stimuli presented at a 10 dB sensation level (SL) relative to the hearing threshold. During the test, two sounds of different frequencies (e.g., 1 vs. 8 kHz) were presented to the ear contralateral to the tinnitus, and the participant indicated which sound was closer to the pitch of their tinnitus. The frequencies were adjusted based on the participant’s responses, with the range being continuously narrowed until the frequency that most closely matched the tinnitus pitch was identified.

Loudness matching (LM) was estimated after determining the frequency that best matched the tinnitus pitch. The stimulus level in the contralateral ear was adjusted to match the loudness of tinnitus, initially using a 5-dB step size and transitioning to a 1-dB step size for finer adjustments. This tinnitus-matching procedure was repeated twice, with a brief break between the two runs. The final adjusted level was averaged across both runs and expressed in terms of dB SL, relative to the hearing threshold of the pitch-matched frequency. Tinnitometry were conducted prior to the MRI scan, which was performed at a separate facility on the same day, with a minimum interval of two hours between assessments. The raw PM and LM data are available in Supplementary Data Sheet 1.

### 2.4 Image acquisition

Imaging was performed using a 3.0 Tesla magnetic resonance scanner equipped with a 15-channel phased-array head coil. Participants were instructed to keep their eyes closed, remain awake, and maintain a neutral mental state. Earplugs and padded clamps were used to reduce scanner noise and minimize head motion. First, 3D T1-weighted images (T1WI) were acquired using a turbo field echo sequence with the following parameters: repetition time (TR)/echo time (TE) = 6.9/3.2 ms, slice thickness = 1 mm, matrix size = 256 × 240, field of view = 256 × 256 × 185 mm, flip angle = 8°, and voxel size = 1 × 1 × 1 mm. These structural images were used for voxel localization and brain-tissue segmentation. Subsequently, single-voxel proton magnetic resonance spectroscopy (^1^H-MRS) was performed using the MEGA-PRESS sequence. The acquisition parameters included TR/TE = 2000/68 ms, spectral bandwidth = 2000 Hz, 2048 data points, and 320 signal averages, with eight unsuppressed water averages. The spectroscopy voxel (4 × 2 × 3 cm^3^; total volume = 24 cm^3^) was positioned in the ACC region on the T1WI for each participant. The ACC was manually delineated on individual T1W images using anatomical landmarks (genu of the corpus callosum anteriorly, anterior commissure posteriorly, cingulate sulcus superiorly, and callosal sulcus inferiorly) in ITK-SNAP. Segmentation aligned with the Palomero-Gallagher atlas (Palomero-Gallagher et al., 2008), and inter-rater reliability was confirmed (ICC = 0.89).

### 2.5 Data preprocessing and quality assessment

**Figure 1:**
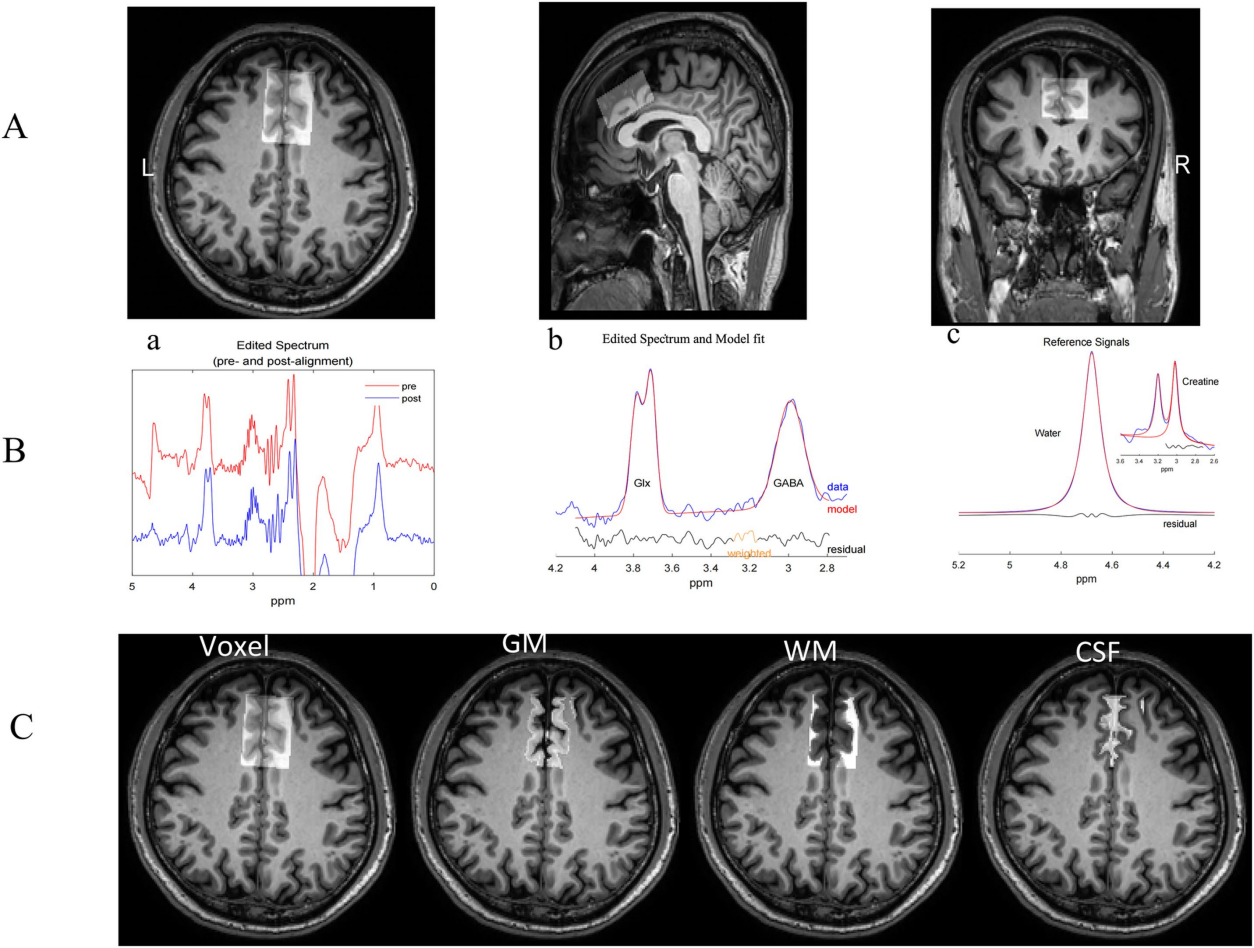
Voxel placement and gannet post-processing modules. **(A)** The voxel location in the anterior cingulate cortex (ACC) is shown using axial, sagittal, and coronal planes (dimensions: 30 × 40 × 20 mm^3^). **(B)** The Gannet Load module displays GABA-edited difference spectra before (red) and after (blue) frequency and phase corrections. (a) The corrected spectra; (b) modeling of the Glx (3.74 ppm) and GABA+ (3.02 ppm) signals, with “Residual” (black) representing the difference between the experimental data (blue) and the fitted model (red); (c) modeling of the creatine (Cr) signal at 3.0 ppm from the OFF spectrum. Data are shown in blue, models in red, and residuals in black. **(C)** The Gannet Segment module illustrates tissue segmentation for the ACC voxel on axial T1-weighted images, including GM, WM, and CSF. L, left; R, right; ACC, anterior cingulate cortex; GABA, gamma-aminobutyric acid; Glx, glutamate/glutamine compounds; Cr, creatine; GM, gray matter; WM, white matter; CSF, cerebrospinal fluid.

Data preprocessing was conducted using Gannet (version 3.0), a MATLAB-based toolbox (Edden et al., 2014). The initial steps involved zero-filling and the application of Gaussian line broadening (3 Hz). Frequency and phase corrections were performed using spectral registration of the individual averages. GABA+ levels were quantified at 3.02 ppm using a nonlinear least-squares fitting approach, accounting for overlapping macromolecular and homocarnosine signals (GABA+; Srinivasan et al., 2005; Shungu et al., 2016). Glutamate and glutamine (Glx) levels, representing the combined signal from glutamate and glutamine, were measured at 3.74 ppm owing to challenges in distinguishing these metabolites at clinical field strength (Arm et al., 2021). Both GABA+ and Glx concentrations were normalized to creatine (Cr) and quantified at 3.0 ppm in the OFF spectrum using the Lorentzian model. The Gannet CoRegister module aligned the spectroscopic voxel with the T1WI, generating binary masks with identical voxel geometries. Tissue segmentation was performed using Gannet Segment in conjunction with SPM12 software.^1^ This step yielded gray matter, white matter, and cerebrospinal fluid fractions, which were incorporated into subsequent analyses. A summary of this process is shown in Figure 1.

### 2.6 Statistical analysis

Shapiro–Wilk tests were used to determine the normality of distribution for variables, If the *p*-value is ≥0.05, indicating normality; if *p* < 0.05, suggesting non-normality. Normally distributed quantitative variables are expressed as mean ± standard deviation, while non-normally distributed data are presented as medians and interquartile ranges. One-way ANOVA, a *t*-test, or the Mann–Whitney U test was used to compare group data. Pearson’s correlations were used to compare demographic and experimental data. Statistical analyses were conducted using IBM SPSS Statistics software (version 27.0; Armonk, NY, USA). In all analyses, statistical significance was defined as *p* < 0.05.

## 3 Results

Overall, one-way analysis of variance (ANOVA) showed no significant differences in age (*F* = 0.395, *p* = 0.675) or PTA (*F* = 1.699, *p* = 0.691) among the RO, CH or NC groups. The Mann–Whitney U test indicated that the duration of tinnitus was significantly shorter in the RO tinnitus group (median = 1 month) than in the CH tinnitus group (median = 18 months) (*Z* = −5.232, *p* < 0.001).

Table 1 presents the demographic and clinical characteristics of participants in the CH and RO tinnitus groups. No significant differences were found between the groups regarding sex distribution (χ^2^ = 0.983, *p* = 0.321), age (*t* = 0.253, *p* = 0.802), or hearing levels as measured using PTA (*t* = −1.355, *p* = 0.184). The duration of tinnitus was significantly shorter in the RO group than in the CH group (*Z* = −5.232, *p* < 0.001). There were no significant differences in PM (*Z* = −0.380, *p* = 0.704), LM (*t* = −1.328, *p* = 0.193), or lateralization patterns (*χ*^2^ = 0.347, *p* = 0.841) between the RO and CH groups. In terms of tinnitus severity, there were no significant differences between the groups in VAS scores (*t* = 0.364, *p* = 0.718) or TFI scores (*t* = −1.141, *p* = 0.261). However, within the TFI subscales, the cognitive subscale score was significantly higher in the RO group than in the CH group (*t* = −2.572, *p* = 0.014), and the auditory subscale score was also significantly elevated in the RO group (*Z* = −1.976, *p* = 0.048). The raw VAS and TFI scores are available in Supplementary Data Sheet 1.

**Table 1:** Demographic and clinical characteristics of CH and RO groups.

	Chronic (*n* = 22)	Recent onset (*n* = 16)	Statistic values	*p*
Sex (male/female)	17/5	10/6	*χ*^2^ = 0.983	0.321
Age (years)	37.2 ± 11.2	36.3 ± 9.4	*t* = 0.253	0.802
PTA (dB HL)	13.5 ± 5.8	15.9 ± 4.8	*t* = −1.355	0.1841
Duration (month)	18 (12, 60)	1 (0.5, 3.0)	*Z* = −5.232	<0.001*
PM (kHz)	6.2 (2.0, 8.0)	5.9 (3.3, 8.0)	*Z* = −0.380	0.704
LM (dB HL)	34.7 ± 19.1	43.0 ± 18.7	*t* = −1.328	0.193
Side (L/R/B)	9/8/5	7/6/3	*χ*^2^ = 0.347	0.841
VAS	4.6 ± 1.4	4.4 ± 1.2	*t* = 0.364	0.718
TFI	34.3 ± 16.1	39.8 ± 12.1	*t* = −1.141	0.261
TFI-I	16.0 ± 6.0	15.4 ± 4.4	*t* = 0.327	0.746
TFI-SC	13.0 ± 5.6	15.1 ± 4.7	*t* = −1.227	0.228
TFI-C	7.0 ± 5.8	10.9 ± 3.6	*t* = −2.572	0.014*
TFI-SL	11.1 ± 8.2	12.9 ± 8.0	*t* = −0.669	0.508
TFI-A	2 (0.8, 9)	7 (3.5, 12.8)	*Z* = −1.976	0.048*
TFI-R	13.3 ± 7.9	13.4 ± 6.5	*t* = −0.023	0.981
TFI-Q	5.5 (0, 14.5)	12 (4.5, 17.8)	*Z* = −1.189	0.234
TFI-E	11.3 ± 6.8	13.4 ± 7.1	*t* = −0.950	0.349

Data are presented as mean ± standard deviation, median (interquartile range), or count, where applicable. PTA, pure-tone average; PM, pitch match; LM, loudness match; L, left ear; R, right ear; B, bilateral; VAS, visual analog scale; TFI, Tinnitus Functional Index; TFI-I, Tinnitus Functional Index–Intrusive; TFI-SC, Tinnitus Functional Index–Sense of Control; TFI-C, Tinnitus Functional Index–Cognitive; TFI-SL, Tinnitus Functional Index–Sleep; TFI-A, Tinnitus Functional Index–Auditory; TFI-R, Tinnitus Functional Index–Relaxation; TFI-Q, Tinnitus Functional Index–Quality of Life; TFI-E, Tinnitus Functional Index–Emotional. ^*^Statistical significance (*p* < 0.05). Statistical tests used include *t*-test, the chi-square test (*χ*^2^), and the Mann–Whitney U test (*Z*).

**Figure 2:**
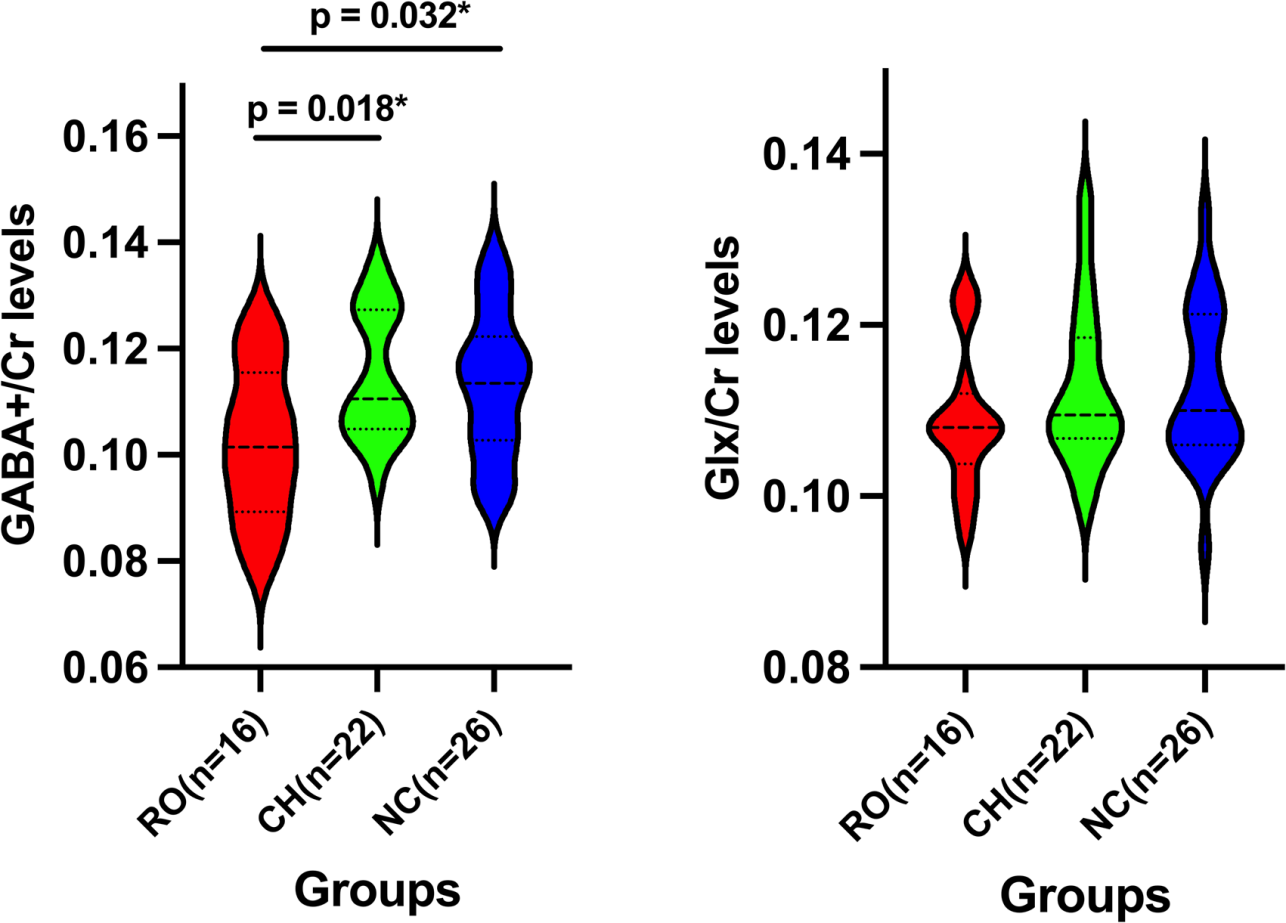
Violin Plots of GABA+/Cr and Glx/Cr Levels Across Three Groups. Violin plots showing the GABA+/Cr and Glx/Cr levels in the three groups: RO tinnitus (red), CH tinnitus (green), and NC (blue). The dashed lines within the violins indicate the median and interquartile range. RO, recent-onset; CH, chronic; NC, normal control; GABA+, gamma-aminobutyric acid; Glx, glutamate/glutamine compounds; Cr, creatine; * indicates *p* < 0.05.

Figure 2 shows the GABA+/Cr and Glx/Cr levels in ACC for the RO tinnitus, CH tinnitus, and NC groups. Raw data for GABA+/Cr and Glx/Cr levels can be found in Supplementary Data Sheet 1. The mean GABA+/Cr levels were 0.102 ± 0.014 for the RO group, 0.115 ± 0.012 for the CH group, and 0.113 ± 0.013 for the NC group. The mean Glx/Cr levels were 0.109 ± 0.008 for the RO group, 0.112 ± 0.009 for the CH group, and 0.113 ± 0.009 for the NC group. Tukey’s multiple comparison tests revealed that the GABA+/Cr levels in the RO group were significantly lower than those in the CH group (*p* = 0.018) and NC group (*p* = 0.032). No significant differences were found for the other comparisons among the three groups (all *p* > 0.05).

Table 2 shows the correlations between clinical characteristics and neurochemical levels (GABA+/Cr and Glx/Cr) in the ACC. A significant positive correlation was found between GABA+/Cr levels and tinnitus duration (*r* = 0.364, *p* = 0.025), suggesting that GABA+/Cr levels increased with longer tinnitus duration. No significant correlations were observed with PTA (r = −0.241, *p* = 0.146), PM (r = 0.039, *p* = 0.818), LM (r = −0.035, *p* = 0.834), or the total TFI (r = 0.079, *p* = 0.637). Additionally, none of the TFI subscales showed significant correlations with GABA+/Cr levels (all *p* > 0.05). Glx/Cr levels were significantly correlated with PM (r = 0.421, *p* = 0.008), indicating that higher Glx/Cr levels were associated with increased pitch match frequencies. A significant positive correlation was also found between Glx/Cr levels and TFI-E scores (r = 0.370, *p* = 0.022), suggesting that higher Glx/Cr levels were linked to greater emotional distress. No significant correlations were found in the remaining analyses (all *p* > 0.05).

**Table 2:** Correlations between clinical characteristics and GABA+/Cr and Glx/Cr levels in ACC.

	GABA+/Cr in ACC		Glx/Cr in ACC	
	*r*	*p*	*r*	*p*
Duration (months)	0.364	0.025*	0.122	0.465
PTA (dB HL)	−0.241	0.146	−0.300	0.068
PM (Hz)	0.039	0.818	0.421	0.008*
LM (dB HL)	−0.035	0.834	0.171	0.306
VAS	0.116	0.489	0.233	0.160
TFI	0.079	0.637	0.232	0.161
TFI-I	0.184	0.268	0.312	0.056
TFI-SC	−0.062	0.712	0.305	0.062
TFI-C	0.055	0.743	0.210	0.207
TFI-SL	0.028	0.865	0.050	0.764
TFI-A	−0.080	0.634	−0.031	0.851
TFI-R	0.242	0.143	0.057	0.732
TFI-Q	0.097	0.562	0.106	0.527
TFI-E	−0.048	0.776	0.370	0.022*

ACC, anterior cingulate cortex; PTA, pure-tone average; PM, pitch match; LM, loudness match; L, left ear; R, right ear; B, bilateral; VAS, visual analog scale; TFI, Tinnitus Functional Index; TFI-I, Tinnitus Functional Index–Intrusive; TFI-SC, Tinnitus Functional Index–Sense of Control; TFI-C, Tinnitus Functional Index–Cognitive; TFI-SL, Tinnitus Functional Index–Sleep; TFI-A, Tinnitus Functional Index–Auditory; TFI-R, Tinnitus Functional Index–Relaxation; TFI-Q, Tinnitus Functional Index–Quality of Life; TFI-E, Tinnitus Functional Index–Emotional. *Uncorrected *p*-values < 0.05. Statistical results are presented as Pearson’s correlation coefficient (*r*) and significance level (*p*).

## 4 Discussion

### 4.1 GABAergic dysregulation in recent-onset tinnitus

This study underscores the critical role of GABAergic neurotransmission in tinnitus, particularly within the ACC. We observed that individuals with RO tinnitus had significantly lower GABA+/Cr levels in the ACC than those with CH tinnitus and normal controls. Interestingly, no significant differences in GABA+/Cr levels were found between CH tinnitus patients and healthy controls. Furthermore, a positive correlation between GABA+/Cr levels and tinnitus duration (r = 0.364, *p* = 0.025) was observed across both the RO and CH tinnitus groups.

The lower GABA+/Cr levels in RO tinnitus patients align with previous research indicating that disruptions in the inhibitory–excitatory balance within the central auditory system contribute to the onset of tinnitus (Leaver et al., 2016; Brozoski et al., 2012). GABA, the primary inhibitory neurotransmitter in the brain, plays a vital role in regulating neuronal excitability. Dysfunction in the ACC likely enhances excitability, which may lead to the perception of phantom auditory stimuli (Qin et al., 2010). As a central hub for sensory, emotional, and cognitive processing, the ACC is especially vulnerable to maladaptive neuroplastic changes during the early stages of tinnitus, which increase cortical reactivity and amplify neural responses to auditory stimuli (Eggermont and Roberts, 2004).

Preclinical research has shown that reduced GABAergic activity in the central auditory pathways, such as the dorsal cochlear nucleus and inferior colliculus, is linked to the neural hyperactivity associated with tinnitus (Middleton et al., 2011; Dong et al., 2010). One recent clinical research performed MRS on 16 tinnitus in two bilateral voxels in the primary auditory cortex to elucidate the role of excitatory and inhibitory neurotransmitters in tinnitus. They reported a lower GABA concentration in the left auditory cortex, indicating a dysfunctional auditory inhibition system in tinnitus (Isler et al., 2022).

The normalization of GABA+/Cr levels in CH tinnitus patients, contrasting with the reduced GABA+/Cr in recent-onset tinnitus, may reflect temporally dynamic processes. Early tinnitus could drive an initial imbalance in ACC inhibition, prompting downstream neuroplastic adaptations. Chronic tinnitus may involve compensation through mechanisms such as strengthened corticolimbic connectivity or shifts in glutamatergic-GABAergic equilibrium, restoring GABA levels to normative ranges despite persistent percepts. Such compensatory plasticity aligns with fMRI studies showing diminished ACC reactivity to tinnitus over time (De Ridder et al., 2014), suggesting reorganization rather than sustained neurotransmitter deficits. One recent structural study has also shown that recent-onset (RO) tinnitus exhibited changes in the topology of brain functional networks when compared to healthy controls. However, as the duration of tinnitus increased, most of these changes recovered (Han et al., 2025). One animal study observed bidirectional and region-specific changes in GABA and glutamate levels in the auditory pathways of rats with CH tinnitus, indicating that significant alterations in inhibitory and excitatory equilibria are associated with this condition (Brozoski et al., 2012). Another animal study on GABAergic function in the auditory thalamus of rats with tinnitus suggests increased tonic GABA_A_R currents and GABA_A_R *δ*-subunit gene expression, which may serve as markers of tinnitus pathology and indicate compensatory mechanisms in the thalamus (Sametsky et al., 2015). Together, these findings support the idea that reorganization rather than sustained neurotransmitter deficits in CH tinnitus patients. This adaptive process likely involves cortical reorganization and the recruitment of alternative brain networks, which may help alleviate the sensory and emotional effects of CH tinnitus (Brozoski et al., 2012).

### 4.2 Glutamatergic activity in tinnitus perception and emotional impact

Our findings also highlight the importance of glutamatergic activity in tinnitus. We observed a positive correlation between Glx/Cr levels and PM frequencies, suggesting that higher glutamatergic activity may correspond to a higher perceptual pitch in tinnitus. Additionally, Glx/Cr levels were positively correlated with TFI-E scores, linking glutamatergic dysfunction to the emotional distress experienced by tinnitus patients. To control for type I error inflation due to multiple comparisons, we applied the Benjamini-Hochberg false discovery rate (FDR) correction (*q* = 0.05) to all correlation tests between neurochemical levels and clinical measures. We found that these findings did not survive strict multiple comparison correction. Associations between Glx/Cr levels and pitch matching/emotional distressshould be interpreted as hypothesis-generating and require replication in larger cohorts.

Previous studies have shown that abnormal glutamatergic activity is often present in the auditory pathways of tinnitus patients. Specifically, increased glutamatergic activity in regions such as the dorsal cochlear nucleus and auditory cortex has been associated with heightened neuronal excitability and the generation of phantom auditory perceptions (Brozoski et al., 2012; Fan et al., 2024; Heeringa et al., 2018; Middleton et al., 2011; Vogler et al., 2011; Xue et al., 2024). Still, there are exceptions. Isler et al. (2022), which study is the first one to measured metabolite levels with MRS in people with tinnitus. They reported an unexpected lower Glu concentration in right auditory cortex. Then Wójcik et al. (2023) investigated metabolite levels in the temporal brain areas excluding primary auditory cortex (complementary to other existing works) in a cohort of 52 tinnitus patients. They failed to demonstrate any significant relationship between tinnitus and Glx/tCr concentration in the temporal brain region of interest which excluded the primary auditory cortex. However, few studies have examined glutamatergic activity in the ACC during tinnitus, despite its significance in the pathophysiology of the condition. Fan et al. (2021) reported a notable increase in spontaneous firing rates and extracellular glutamate levels in the ACC in models of sodium salicylate–induced tinnitus, which were associated with altered neural activity patterns such as reduced alpha band activity and increased beta and gamma band activity.

For the first study, which measured glutamate concentration in ACC with MRS in tinnitus patients, we did not reveal significant differences in ACC Glx/Cr levels between tinnitus patients and healthy controls. This discrepancy may be attributed to several factors, including differences in study design, sample characteristics, and methodological approaches. For example, this study included tinnitus patients without hearing loss, while many previous studies have focused on individuals with varying degrees of hearing impairment. Hearing loss triggers plastic changes in the auditory system (Balaram et al., 2019), such as altered vesicular glutamate transporter distribution and increased glutamatergic activity in the cochlear nucleus and central auditory pathways. These changes are thought to contribute to tinnitus through compensatory mechanisms that enhance neuronal activity (Zeng et al., 2009). Additionally, the paraflocculus of the cerebellum is involved in regulating whether this increased auditory activity translates into conscious tinnitus perception, suggesting a complex interaction between auditory and non-auditory brain regions (Mulders et al., 2014). Importantly, the mechanisms of tinnitus differ significantly between individuals with and without hearing loss. The excitatory and inhibitory neurotransmission changes, particularly those involving glutamatergic signaling, create distinct neural environments for tinnitus in people with normal hearing versus those with hearing loss (Eggermont and Roberts, 2004). Therefore, excluding participants with hearing loss from tinnitus studies helps clarify the neural mechanisms specific to tinnitus, minimizing confounding factors related to hearing loss. This approach enables researchers to pinpoint unique pathways and biomarkers of tinnitus in normal-hearing populations, enhancing the specificity of the findings and reducing variability.

### 4.3 Limitations

While this study offers valuable insights into the neurochemical basis of tinnitus, there are several limitations that warrant consideration. Firstly, the cross-sectional design prevents causal inferences about neurochemical changes over time. Longitudinal studies are needed to track the progression of these changes from the onset of tinnitus to its chronic phase. Secondly, emerging evidence highlights functional heterogeneity across ACC subdivisions. For instance, Vanneste et al. (2024) recently demonstrated that gamma-band oscillations in the dorsal ACC (dACC) correlate with tinnitus severity, whereas pregenual ACC (pgACC) connectivity with the default mode network (DMN) predicts maladaptive rumination. MRS requires a relatively large voxel size to reliably detect low-concentration metabolites like GABA, which limits anatomical specificity (e.g., pgACC, rACC, dACC). Consequently, our GABA and glutamate measures likely reflect averaged neurochemistry across ACC subregions and neighboring tissues, obscuring subdivision-specific imbalances. Future studies should combine ultra-high-field MRS (e.g., 7 T) with advanced segmentation protocols to resolve these limitations. Thirdly, our sample size (*n* = 16 for resent-onset tinnitus, *n* = 22 for chronic tinnitus, *n* = 26 for normal control) was constrained by the rigorous recruitment criteria (e.g., narrow tinnitus chronicity window, normal hearing thresholds), which prioritized homogeneity at the cost of reduced generalizability. Although comparable to prior MRS studies in tinnitus (e.g., *n* = 16 for tinnitus and *n* = 17 for normal control in Isler et al., 2022), the absence of formal *a priori* power calculations limits definitive conclusions, particularly for subgroup analyses. Future studies with larger cohorts are warranted to validate these associations. Fourthly, note that the “normal hearing”designation in this study should be interpreted with caution, as standard pure-tone thresholds (0.25–8 kHz) do not preclude the presence of hidden cochlear synaptopathy or other subclinical auditory pathologies (Liberman et al., 2016). Future studies incorporating electrophysiological measures (e.g., ABR wave I amplitude, ECochG) are needed to clarify HHL’s role in tinnitus neurochemistry. Finally, emerging evidence suggests that psychiatric comorbidities such as anxiety and depression may modulate GABAergic tone in the ACC (Sanacora et al., 2004; Hasler et al., 2007; Bhagwagar et al., 2008). While our tinnitus cohort did not self-report clinically diagnosed mood disorders, we lacked formal psychometric evaluations to rule out subclinical anxiety or depression. Future studies should include standardized psychiatric assessments to disentangle tinnitus-specific neurochemistry from overlapping mood-related alterations.

## 5 Conclusion

This study delineates temporally distinct neurochemical dynamics in the anterior cingulate cortex (ACC) across tinnitus progression, revealing an early GABAergic deficit in recent-onset patients that normalizes with chronicity, suggesting compensatory restoration of inhibitory tone over time. While glutamatergic activity did not differ between groups, exploratory correlations implicated heightened Glx/Cr in perceptual (pitch) and affective (emotional distress) dimensions of tinnitus, warranting further investigation. These findings position ACC GABAergic dysregulation as a potential therapeutic target during critical early stages of tinnitus and underscore the need for longitudinal studies to unravel the molecular cascades driving neurochemical recalibration.

## Data Availability

The datasets presented in this study can be found in online repositories. The names of the repository/repositories and accession number(s) can be found in the article/Supplementary material.

## Footnotes

^1^ http://www.fil.ion.ucl.ac.uk/spm/
